# Household smoking and dental caries in schoolchildren: the Ryukyus Child Health Study

**DOI:** 10.1186/1471-2458-10-335

**Published:** 2010-06-14

**Authors:** Keiko Tanaka, Yoshihiro Miyake, Masashi Arakawa, Satoshi Sasaki, Yukihiro Ohya

**Affiliations:** 1 Department of Public Health, Faculty of Medicine, Fukuoka University, Fukuoka, Japan; 2 Field Science for Health and Recreation, Faculty of Tourism Sciences and Industrial Management, University of the Ryukyus, Okinawa, Japan; 3 Department of Social and Preventive Epidemiology, School of Public Health, The University of Tokyo, Tokyo, Japan; 4 Division of Allergy, Department of Medical Specialties, National Center for Child Health and Development, Tokyo, Japan

## Abstract

**Background:**

Secondhand smoke exposure (SHSe) is perhaps one of the most important toxic exposures in childhood. However, epidemiological studies on the relation between SHSe and dental caries are limited and have yielded inconsistent results. The present cross-sectional study examined the potential association between SHSe at home and the prevalence of dental caries in children.

**Methods:**

Subjects were 20,703 schoolchildren aged 6 to 15 years in Okinawa, Japan. Information on SHSe at home and potential confounding factors was obtained through questionnaires. Data on dental caries were obtained from school records. Children were classified as having decayed and/or filled teeth (DFT) if a dentist diagnosed these conditions. Additionally, we analyzed decayed teeth (DT) and filled teeth (FT) separately. Adjustment was made for sex, age, region of residence, toothbrushing frequency, use of fluoride, sugar intake, and paternal and maternal educational level.

**Results:**

The prevalence of DFT was 82.0%. Compared with never smoking in the household, former and current household smoking were independently associated with an increased prevalence of DFT (adjusted prevalence ratios [95% confidence intervals] for former household smoking and current light and heavy household smoking were 1.03 [1.00-1.05], 1.04 [1.02-1.05], and 1.04 [1.03-1.06], respectively); when analyzed separately there was an increased prevalence of DT (adjusted prevalence ratios [95% confidence intervals] for former household smoking and current light and heavy household smoking were 1.06 [1.02-1.11], 1.10 [1.06-1.13], and 1.10 [1.07-1.14], respectively) but not FT. A statistically significant dose-response relationship between cumulative smoking in the household and the prevalence of DFT and DT (*P *for trend < 0.0001), but not FT, was observed. In an analysis of 2 subgroups, subjects who had at least 1 deciduous tooth and subjects who had at least 1 permanent tooth, household smoking exposure was associated with an increased prevalence of DFT and DT not only in those with deciduous but also those with permanent dentition.

**Conclusion:**

Our findings suggested that household smoking might be associated with an increased prevalence of dental caries in children.

## Background

Secondhand smoke exposure (SHSe) is perhaps one of the most important toxic exposures in childhood. Such exposure has been associated with middle-ear diseases, asthma, wheeze, cough, bronchitis, pneumonia, and impaired pulmonary function in children [1,2]. Several studies have reported an association between SHSe and dental caries. In US children aged 4 to 11 years, a positive association between serum cotinine levels and the prevalence of caries in deciduous, but not in permanent teeth, was observed [3]. Data from the UK National Diet and Nutrition Survey showed that maternal smoking was independently associated with an increased prevalence of caries in deciduous teeth among preschool children [4]. A cross-sectional study in Belgium demonstrated that parental smoking was significantly positively associated with the prevalence of caries among 5-year-old children; however, such a relationship was not observed in 3-year-old children [5]. In a cross-sectional study in South Africa, residing with a smoker was significantly positively associated with the prevalence of caries on second molars in adolescents [6]. Smoking in the household was shown to be associated with a higher prevalence of untreated decayed teeth, but not caries experience (decayed and/or filled teeth) in a cross-sectional study among Japanese children aged 1 to 14 years [7]. Thus, epidemiologic evidence of an association between passive smoking and dental caries is inconsistent. From the viewpoint of preventing dental caries in children, it is important to clarify the possible effect of passive smoking on the development of dental caries. Therefore, we examined the potential association between household smoking and dental caries using the data set of the Ryukyus Child Health Study (RYUCHS), which involved a large sample size and collected information on important factors in relation to dental caries and smoking, and school records of dental examinations.

## Methods

### Study population

The RYUCHS, a cross-sectional school-based study, investigated the associations between various selected factors and child health problems. Details of the RYUCHS have been described elsewhere [8,9]. During the period from September 2004 to January 2005, all 35 public elementary schools and 17 junior high schools in Naha City, the largest city in Okinawa Prefecture with a total population of almost 316,000 (population density: about 8,000 persons/km^2^), and 17 public elementary schools and 8 junior high schools in Nago City, which has a total population of almost 60,000 (population density: about 290 persons/km^2^), participated in the RYUCHS. Teachers distributed a set of 2 self-administered questionnaires to all 38,212 children at school. These were completed at home by parents of the elementary schoolchildren and by the junior high school students themselves and/or their parents. Finally, 28,885 sets of questionnaires were returned (participation rate = 75.6%). Permission to perform the RYUCHS was obtained from the ethics committee of the Faculty of Medicine, Fukuoka University.

### Questionnaires

One of the questionnaires included questions about sex, age, dental health behavior, such as toothbrushing frequency and use of fluoride, paternal and maternal educational level, and smoking habits of adult household members. Use of fluoride was defined as positive if children reported using fluoride agents, such as toothpaste, gel and mouthwash. The second questionnaire was a brief self-administered diet history questionnaire (BDHQ). Estimates of daily intake for sugar and energy were calculated using an ad hoc computer algorithm for the BDHQ. Value of sugar intake was energy-adjusted by density methods (g/1,000 kcal).

### Oral examination

In Japan, schoolchildren receive an annual oral examination under the School Health Law. A school dentist performs the oral examination by direct inspection. Oral measurements include the number of total teeth present together with that of treated and untreated teeth and assessment of the gingival condition and oral hygiene status. Radiographs are not taken. These data are forwarded to the Municipal Board of Education from each school. Data on dental examinations performed in 2004 were obtained from the Naha City Municipal Board of Education and the Nago City Municipal Board of Education. We combined data from the oral examinations and the RYUCHS. Of the 28,885 children who took part in the RYUCHS, data on 27,202 were successfully linked. A total of 6499 children were excluded because of missing or illogical data on variables under study, leaving data on 20,703 children available for analysis (54.2% of all eligible children). Children were classified as having decayed and/or filled teeth (DFT) if a dentist diagnosed these conditions. Additionally, we analyzed decayed teeth (DT) and filled teeth (FT) separately. The categories were not mutually exclusive because a child could have both decayed and filled teeth. The present study was approved by the personal information protection committees of the Government of Naha City and the Government of Nago City.

### Statistical analysis

Age was classified into 3 categories (6-8, 9-11, and 12-15 years), region of residence into 2 (Naha City and Nago City), toothbrushing frequency into 4 (<1, 1, 2, and ≥3 times per day), use of fluoride into 2 (yes and no), paternal and maternal educational level into 4 (junior high school, high school, junior college or vocational technical school, and university), smoking in the household into 4 (never, former, and current smoking into <15 or ≥15 cigarettes per day), and pack-years of passive smoking in the household into 4 (none, 0.1-2.9, 3.0-6.9, and ≥7.0 pack-years). Intake of sugar was categorized by quartiles.

Prevalence ratios (PRs) and 95% confidence intervals (CIs) were estimated by binomial regression with the log link function [10-12]. We took into consideration clustering within schools via the PROC GENMOD procedure. To examine whether prevalence increased according to the status of smoking in the household and pack-years of SHSe at home, trends of association were assessed using a log-binomial regression model assigning consecutive integers to the levels of the independent variable. Two-sided *P *values less than 0.05 were considered statistically significant. All statistical analyses were performed by using the SAS software package version 9.1 (SAS Institute, Inc., Cary, NC, USA).

## Results

Of the 20,703 children, 82.0% had DFT, 48.4% had DT, and 68.9% had FT. Mean number of DFT, DT, and FT were 4.3, 1.6, and 2.7, respectively. Toothbrushing twice or more per day was reported by 14,619 (70.6%) children (Table 1). A total of 8623 children (41.7%) were exposed to household smoking at the time of the survey, whereas half of the children (49.2%) had never been exposed to household smoking up to the time of the survey. Among participants, 9.1% were from households with former smokers.

**Table 1:** Distribution of selected characteristics in 20,703 schoolchildren, RYUCHS, Japan

Variable	n (%)
Male sex	10,201 (49.3)
Age (y)	
6-8	6,088 (29.4)
9-11	7,522 (36.3)
12-15	7,092 (34.3)
Region of residence	
Nago city	3,540 (17.1)
Naha city	17,163 (82.9)
Toothbrushing frequency (times/day)	
<1	1,566 (7.6)
1	4,518 (21.8)
2	11,925 (57.6)
≥3	2,694 (13.0)
Use of fluoride	
No	4,531 (21.9)
Yes	16,172 (78.1)
Paternal educational level	
Junior high school	1,540 (7.4)
High school	9,160 (44.2)
Junior college or vocational technical school	3,097 (15.0)
University	6,906 (33.4)
Maternal educational level	
Junior high school	1,024 (5.0)
High school	9,071 (43.8)
Junior college or vocational technical school	8,704 (42.0)
University	1,904 (9.2)
Daily sugar intake (g/1,000 kcal)	
<3.0200	5,176 (25.0)
3.0200-4.6510	5,176 (25.0)
4.6511-6.8298	5,176 (25.0)
≥6.8299	5,175 (25.0)

Table 2 shows the results of bivariate analyses for selected covariates. Frequent toothbrushing was associated with a lower prevalence of decayed and/or filled teeth. Higher paternal and maternal educational levels were associated with a lower prevalence of decayed and/or filled teeth; children having fathers with a university degree or having mothers with a university degree were more unlikely to experience caries than the reference groups. There was a stepwise increase in the prevalence of decayed and/or filled teeth in relation to daily sugar intake; prevalence was highest among children in the highest quartile.

**Table 2:** Prevalence ratios (PRs) and 95% confidence intervals (CIs) for decayed and/or filled teeth in relation to study variables

	Prevalence (%)	Crude PR (95% CI)
Sex		
Female	8624/10502 (82.1)	1.00
Male	8348/10201 (81.8)	1.00 (0.98-1.01)
Age (y)		
6-8	4989/6088 (81.5)	1.00
9-11	6301/7522 (83.8)	1.03 (1.01-1.04)
12-15	5712/7093 (80.5)	0.99 (0.97-1.01)
Region of residence		
Nago city	3014/3540 (85.1)	1.00
Naha city	13958/17163 (81.3)	0.96 (0.94-0.97)
Toothbrushing frequency (times/day)		
<1	1372/1566 (87.6)	1.00
1	3823/4518 (84.6)	0.97 (0.94-0.99)
2	9688/11925 (81.2)	0.93 (0.91-0.95)
≥3	2089/2694 (77.5)	0.89 (0.86-0.91)
Use of fluoride		
No	3705/4531 (81.8)	1.00
Yes	13267/16172 (82.0)	1.00 (0.99-1.02)
Paternal educational level		
Junior high school	1357/1540 (88.1)	1.00
High school	7740/9160 (84.5)	0.96 (0.94-0.98)
Junior college or vocational technical school	2518/3097 (81.3)	0.92 (0.90-0.95)
University	5357/6906 (77.6)	0.88 (0.86-0.90)
Maternal educational level		
Junior high school	900/1024 (88.0)	1.00
High school	7654/9071 (84.4)	0.96 (0.94-0.98)
Junior college or vocational technical school	6948/8704 (79.3)	0.91 (0.89-0.93)
University	1473/1904 (77.4)	0.88 (0.85-0.91)
Daily sugar intake (g/1,000 kcal)		
<3.0200	4054/5176 (78.3)	1.00
3.0200-4.6510	4192/5176 (81.0)	1.03 (1.01-1.05)
4.6511-6.8298	4335/5176 (83.8)	1.07 (1.05-1.09)
≥6.8299	4391/5175 (84.9)	1.08 (1.06-1.10)

In an overall analysis, compared with never smoking in the household, former and current smoking in the household were significantly associated with an increased prevalence of DFT and, when analyzed separately, with the presence of DT, but not FT (Table 3). After adjustment for sex, age, region of residence, toothbrushing frequency, use of fluoride, sugar intake, and paternal and maternal educational level, these positive associations were slightly attenuated but remained statistically significant. The adjusted means of DFT were 4.1 and 4.5 among those in never-smoking households and those exposed to smoke from at least 15 cigarettes a day at home, respectively (*P *for trend < 0.0001). Statistically significant dose-response relationships of cumulative passive smoking at home to the prevalence of DFT and DT were observed in the multivariate model (*P *for trend < 0.0001). No significant dose-response association with the prevalence of FT was observed, although 0.1 to 2.9 pack-years of household smoking was significantly positively associated with the prevalence of FT. The number of pack-years of passive smoking in the household was significantly positively related to DFT and DT. The adjusted means of DFT and DT were 4.5 and 1.8, respectively, among those in households with 7.0 or more pack-years of passive smoking (*P *for trend < 0.0001).

**Table 3:** Crude and adjusted prevalence ratios (PRs) and 95% confidence intervals (CIs) for dental caries in relation to smoking in the household of 20,703 schoolchildren, RYUCHS, Japan

	Prevalence (%)	Crude PR (95% CI)	Adjusted PR^1 ^(95% CI)
**Decayed and/or filled teeth**			
Smoking in household			
Never	8080/10192 (79.3)	1.00	1.00
Former	1574/1888 (83.4)	1.05 (1.03, 1.08)	1.03 (1.00, 1.05)
Current <15^2^	4403/5215 (84.4)	1.07 (1.05, 1.08)	1.04 (1.02, 1.05)
Current ≥15^2^	2915/3408 (85.5)	1.08 (1.06, 1.10)	1.04 (1.03, 1.06)
*P *for trend		< 0.0001	< 0.0001
Pack-years of passive smoking in household			
None	8080/10192 (79.3)	1.00	1.00
0.1-2.9	2827/3352 (84.3)	1.06 (1.05, 1.08)	1.03 (1.02, 1.05)
3.0-6.9	2935/3490 (84.1)	1.06 (1.04, 1.08)	1.03 (1.01, 1.05)
≥7.0	3130/3669 (85.3)	1.08 (1.06, 1.09)	1.04 (1.03, 1.06)
*P *for trend		< 0.0001	< 0.0001
**Decayed teeth**			
Smoking in household			
Never	4394/10192 (43.1)	1.00	1.00
Former	944/1888 (50.0)	1.16 (1.10, 1.22)	1.06 (1.02, 1.11)
Current <15^2^	2797/5215 (53.6)	1.24 (1.20, 1.29)	1.10 (1.06, 1.13)
Current ≥15^2^	1889/3408 (55.4)	1.29 (1.24, 1.33)	1.10 (1.07, 1.14)
*P *for trend		<0.0001	<0.0001
Pack-years of passive smoking in household			
None	4394/10192 (43.1)	1.00	1.00
0.1-2.9	1717/3352 (51.2)	1.19 (1.14, 1.24)	1.06 (1.03, 1.10)
3.0-6.9	1943/3490 (55.7)	1.29 (1.24, 1.34)	1.13 (1.10, 1.17)
≥7.0	1790/3669 (53.7)	1.25 (1.20, 1.29)	1.11 (1.08, 1.15)
*P *for trend		<0.0001	<0.0001
**Filled teeth**			
Smoking in household			
Never	6974/10192 (68.4)	1.00	1.00
Former	1305/1888 (69.1)	1.01 (0.98, 1.04)	1.00 (0.97, 1.04)
Current <15^2^	3623/5215 (69.5)	1.02 (0.99, 1.04)	1.01 (0.99, 1.04)
Current ≥15^2^	2362/3408 (69.3)	1.01 (0.99, 1.04)	1.01 (0.98, 1.04)
*P *for trend		0.18	0.31
Pack-years of passive smoking in household			
None	6974/10192 (68.4)	1.00	1.00
0.1-2.9	2376/3352 (70.9)	1.04 (1.01, 1.06)	1.03 (1.01, 1.06)
3.0-6.9	2369/3490 (67.9)	0.99 (0.97, 1.02)	0.99 (0.96, 1.02)
≥7.0	2545/3669 (69.4)	1.01 (0.99, 1.04)	1.01 (0.98, 1.03)
*P *for trend		0.55	0.93

^1 ^Adjustment for sex, age, region of residence, toothbrushing frequency, use of fluoride, sugar intake, and paternal and maternal educational level.

^2 ^Number of cigarettes smoked in the household per day.

To estimate separately the association between household smoking and the prevalence of caries in deciduous and permanent dentition, we analyzed 2 subgroups: subjects who had 1 or more deciduous teeth (deciduous subgroup, n = 13,872) and subjects who had 1 or more permanent teeth (permanent subgroup, n = 20,253) (Table 4). Children with mixed dentition were assigned to both groups. In the deciduous subgroup, compared with never household smoking, current household smoking was independently positively associated with the prevalence of DFT, DT, and FT. A significant positive dose-response relationship between pack-years of passive smoking in the household and the prevalence of DFT and DT was observed (*P *for trend < 0.0001). For FT in the deciduous subgroup, there was a tendency for a positive dose-response trend (*P *for trend = 0.06). For the permanent subgroup, current household smoking was significantly associated with an increased prevalence of DFT and DT, but not FT. Significant positive dose-response relationships were observed between pack-years of passive smoking in the household and the prevalence of DFT and DT, but not FT. Positive significant trends for the adjusted means for DFT and DT across the four categories of household smoking status and cumulative passive smoking at home were observed in both the deciduous and permanent subgroups.

**Table 4:** Adjusted prevalence ratios (PRs) and 95% confidence intervals (CIs) for dental caries in relation to smoking in the household of 20,703 schoolchildren, RYUCHS, Japan

	Deciduous subgroup (n = 13,872)	Permanent subgroup (n = 20,253)
	
	Adjusted PR^1 ^(95% CI)	Adjusted PR^1 ^(95% CI)
**Decayed and/or filled teeth**		
Smoking in household		
Never	1.00	1.00
Former	1.02 (0.99, 1.06)	1.03 (1.00, 1.06)
Current <15^2^	1.04 (1.02, 1.06)	1.05 (1.02, 1.07)
Current ≥15^2^	1.06 (1.04, 1.08)	1.03 (1.00, 1.06)
*P *for trend	< 0.0001	0.01
Pack-years of passive smoking in household		
None	1.00	1.00
0.1-2.9	1.03 (1.01, 1.06)	1.05 (1.02, 1.08)
3.0-6.9	1.05 (1.03, 1.07)	1.02 (0.99, 1.05)
≥7.0	1.05 (1.03, 1.08)	1.04 (1.01, 1.07)
*P *for trend	<0.0001	0.006
**Decayed teeth**		
Smoking in household		
Never	1.00	1.00
Former	1.06 (1.00, 1.12)	1.09 (1.01, 1.17)
Current <15^2^	1.08 (1.04, 1.12)	1.19 (1.13, 1.25)
Current ≥15^2^	1.12 (1.07, 1.16)	1.16 (1.10, 1.23)
*P *for trend	< 0.0001	< 0.0001
Pack-years of passive smoking in household		
None	1.00	1.00
0.1-2.9	1.04 (1.00, 1.09)	1.12 (1.06, 1.20)
3.0-6.9	1.12 (1.07, 1.17)	1.18 (1.12, 1.26)
≥7.0	1.11 (1.06, 1.16)	1.17 (1.11, 1.24)
*P *for trend	<0.0001	<0.0001
**Filled teeth**		
Smoking in household		
Never	1.00	1.00
Former	1.00 (0.95, 1.04)	1.01 (0.96, 1.06)
Current <15^2^	1.03 (1.00, 1.06)	0.99 (0.96, 1.03)
Current ≥15^2^	1.05 (1.01, 1.09)	0.97 (0.93, 1.01)
*P *for trend	0.003	0.19
Pack-years of passive smoking in household		
None	1.00	1.00
0.1-2.9	1.04 (1.01, 1.07)	1.02 (0.98, 1.07)
3.0-6.9	1.02 (0.98, 1.05)	0.96 (0.92, 1.00)
≥7.0	1.04 (1.00, 1.08)	0.99 (0.96, 1.03)
*P *for trend	0.06	0.26

^1 ^Adjustment for sex, age, region of residence, toothbrushing frequency, use of fluoride, sugar intake, and paternal and maternal educational level.

^2 ^Number of cigarettes smoked in the household per day.

## Discussion

In this present study, household smoking was independently associated with an increased prevalence of the experience of dental caries in Japanese children. Our results are in agreement with previous studies that found a positive relationship between SHSe and dental caries in deciduous teeth [3,4,13], but at variance with a study in Japan showing no association [7]. In the cited cross-sectional study in Japan, the crude information on SHSe (presence of a smoker in the home) and low statistical power due to the small sample size (n = 925) might have made it difficult to detect a significant positive association with SHSe. The present study provided further evidence that exposure to household smoking is associated with an increased prevalence of caries experience not only in deciduous but also in permanent dentition. To our knowledge, only 1 cross-sectional study showed a positive association between SHSe and caries in permanent teeth [6]. That research examined the relationship between residing with a smoker and the prevalence of caries on second molars.

We have no immediate explanation for the potential mechanisms underlying our observations. Exposure to tobacco smoke, which contains numerous chemical toxins, might predispose children to infection through suppression or modulation of the immune system [1]. Numabe *et al. *showed that the phagocytic activity of salivary polymorphonuclear leukocytes intensifies after cigarette smoking and SHSe [14]. An *in vitro *study demonstrated that nicotine inhibited phagocytic activity of neutrophils and monocytes [15]. As dental caries result from chronic bacterial infection in the oral cavity, a potential pathway linking SHSe and caries might be attributed to alterations in host responses.

Environmental tobacco smoke contains lower doses of the same toxins that a smoker inhales, so SHSe might affect oral health through the same mechanisms as with active smoking. Sakki and Knuuttila showed that tobacco smoking was associated with elevated levels of mutans streptococci and lactobacilli [16], which are associated with the initiation and progression of dental caries, in saliva. Among Swedish young adults, smoking habits contributed to low buffering capacity of saliva, which is a protective factor against dental caries [17]. On the other hand, epidemiological studies on the association between active smoking and dental caries showed inconsistent results. Some studies suggested that smoking was positively associated with dental caries [18,19], while another study reported no association between smoking and dental caries [20]. The heterogeneity of these results among studies might be explained by differences in characteristics, smoking habits, and lifestyle of the populations examined, the study design used, and potential confounders considered. In particular, few of the studies on the association between smoking and dental caries controlled for potential confounders, such as socioeconomic status, diet, and oral health behaviors.

Another possible explanation is that some unknown factors related to household smoking may have confounded the observed relationship. A British cross-sectional study showed that teenagers living with parents who smoked had a lower intake of fiber, vitamins C and E, folates, and magnesium than those with parents who were non-smokers [21]. Hu *et al. *demonstrated that smoking habits were significantly positively associated with irregular breakfast eating [22]. In a cross-sectional study of dental students, smokers were less likely to brush their teeth than nonsmokers [23]. It was also observed that regular smokers were more likely to be very afraid of visiting a dentist than those who smoked occasionally or who never smoked [24]. Thus, smokers are likely to have poor dietary habits, an unhealthy lifestyle, and low oral health awareness, which might affect the oral health of their children. In the current study, we found a positive association between SHSe and DT, but not FT, which is indeed not only an indicator of caries experience, but also a reflection of access to or utilization of restorative care that may not be related to SHSe.

Although our results showed that household smoking was positively associated with the prevalence of dental caries, the strength of the observed associations was quite small. Dental caries is a multifactorial disease and is affected by physical, biological, environmental, and lifestyle-related factors, such as a high number of cariogenic bacteria, inadequate salivary flow, insufficient fluoride exposure, and poor oral hygiene [25]. The effect of SHSe on dental caries might be small.

This study has several strengths, which included the large sample size and adjustment for potentially important factors related to dental caries, such as oral hygiene practices, fluoride exposure, sugar intake, and socioeconomic characteristics. However, residual confounding effects could not be ruled out. In particular, information on socioeconomic status was based on only paternal and maternal educational levels. Part of the observed positive relationships between SHSe and dental caries may be ascribed to uncontrolled for confounding effects of socioeconomic status. Data on dental caries taken from school records are likely to avoid information bias. The school dentists were given detailed criteria for the examination, but calibration was not performed among school dentists. Therefore, it was unknown if intra- and interexaminer consistency was established. Additionally, radiographs were not taken. Oral examinations without radiographic information are likely to underestimate the prevalence of dental caries, especially proximal caries [26,27].

Other limitations may also influence the interpretation of the current results. As this study is cross-sectional, the temporal nature of the association between household smoking and dental caries could not be examined. SHSe was assessed by questionnaire reports and was not validated by measurements of biomarkers, such as salivary, serum or urine cotinine levels. Using questionnaires may result in misclassification from recall bias and response bias due to parents' feelings of guilt for smoking in the presence of their children. However, differential misclassification is unlikely because those completing the questionnaires might not be aware of the effect of SHSe on dental caries. The consequence would bias the results toward the null. In addition, responders differed between elementary schools and junior high schools. Parents of elementary school children completed our questionnaires whereas with junior high school students the questionnaires were completed by either the students themselves with or without parental help or by their parents. Accuracy of the replies might differ according to whether parents or children completed the questionnaire. The misclassification of exposure would have given rise to an underestimation of our findings. In the present study, we did not take active smoking among children into account because data on active smoking were collected only among junior high school students. The prevalence of active smoking among junior high school students was very low (2%). When sensitivity analysis excluded these active smokers, the results were not changed, however (data not shown).

Only slightly over half of eligible children (54.2%) were included in this analysis, leading to a potential for selection bias. No information on factors examined was available for the 9327 non-participants in the RYUCHS. Compared with children who were excluded due to incomplete information on the questionnaires, study subjects were more likely to be male, live in Naha city, brush their teeth, experience fluoride application, have family members who had never smoked, and have parents with a high educational level and were less likely to be young, have DFT, and have a higher intake of sugar (data not shown). The loss of children who were at higher risk for caries and who had parents with a lower educational level might lead to underestimation of the strength of the association between household smoking and dental caries. When we analyzed study subjects separately according to city, the results for Naha city were similar to those in the overall analysis. Likewise, the results for Nago city were similar to those in the overall analysis. With regard to testing for statistical significance, the observed significant positive relationships might be ascribed to a Type I error due to the large sample size.

## Conclusion

Our results revealed that SHSe is independently associated with dental caries in Japanese children. Although the biological and behavioral plausibility for an etiological relationship between SHSe and dental caries is likely to be high, epidemiological evidence is still insufficient. The addition of objective markers of SHSe may help to clarify the role of SHSe on dental caries. In addition, prospective studies are needed to identify SHSe as a direct risk factor for the development of caries in children.

## Competing interests

The authors declare that they have no competing interests.

## Authors' contributions

All authors contributed to the study concept and design and the acquisition of data. KT and YM were responsible for the analysis and interpretation of data and the drafting of the manuscript. All authors participated in critically revising the manuscript and approved the final version of the manuscript.

## Pre-publication history

The pre-publication history for this paper can be accessed here:

http://www.biomedcentral.com/1471-2458/10/335/prepub
